# What’s new in melanoma? Combination!

**DOI:** 10.1186/s12967-015-0582-1

**Published:** 2015-07-04

**Authors:** Paolo A Ascierto, Francesco M Marincola, Michael B Atkins

**Affiliations:** Unit of Melanoma, Cancer Immunotherapy and Innovative Therapy, Istituto Nazionale per lo Studio e la Cura dei Tumori “Fondazione G. Pascale”, Via Mariano Semmola, 80131 Naples, Italy; Sidra Medical and Research Centre, Doha, Qatar; Oncology and Medicine, Georgetown-Lombardi Comprehensive Cancer Center, Georgetown University School of Medicine, 3970 Reservoir Rd NW, Washington, DC 20057 USA

## Abstract

Melanoma was again a focus of attention at the 2015 American Society of Clinical Oncology (ASCO) Annual Meeting, in particular the use of combination treatment strategies involving immunotherapies and/or targeted agents. New data on targeted therapies confirmed previous findings, with combined BRAF inhibitor (vemurafenib) plus MEK inhibitor (cobimetinib) improving progression-free survival (PFS) compared to vemurafenib monotherapy in patients with BRAFV600 mutation-positive tumors (CoBRIM trial). Positive results were also seen with combined dabrafenib and trametinib in patients with BRAF V600E/K metastatic melanoma and encorafenib plus binimetinib in BRAFV600-mutant cutaneous melanoma. Even more interesting news centered on the use of combination immunotherapy, in particular the randomized, double-blind CheckMate 067 study in which median PFS with nivolumab plus ipilimumab was 11.5 months, compared to 2.9 months with ipilimumab alone (HR 0.42) and 6.9 months with nivolumab alone (HR 0.57). Of interest, in patients with ≥5% PD-L1 expression, median PFS was 14 months with the combination or with nivolumab alone compared with 3.9 months in the ipilimumab group, while in the PD-L1 negative cohort, the combination remained superior to both monotherapies. Given that combination therapy was accompanied by a high occurrence of side-effects, this raises the suggestion that combination therapy might be reserved for PD-L1 negative patients only, with PD-L1 positive patients achieving the same benefit from nivolumab monotherapy. However, overall survival data are awaited and the equivalence of single agent to the combination remains unconvincing. Interesting data were also reported on the combination of T-VEC (talimogene laherparepvec) with ipilimumab, and the anti-PD-1 agent MEDI4736 (durvolumab) combined with dabrafenib plus trametinib. Emerging data also suggested that predictive markers based on immunoprofiling and mismatch repair deficiency may be of clinical use. In conclusion, the use of combination approaches to treat patients with melanoma, as well as other cancers, is no longer a just a wish for the future but is today a clinical reality with a rapidly growing evidence-base. Moreover, the most exciting consideration is that this is far from the end of the story, but rather a fantastic introduction.

As in recent years, melanoma was a focus of attention at the 2015 American Society of Clinical Oncology (ASCO) Annual Meeting. If a single word could sum up this years’ melanoma news from ASCO, then “combination” would surely be the most appropriate.

New data were reported on targeted therapies, confirming the excellent results previously reported [1, 2]. An update on the CoBRIM trial of combined BRAF inhibitor (vemurafenib) plus MEK inhibitor (cobimetinib) in patients with BRAFV600 mutation-positive tumors confirmed its superior impact on progression-free survival (PFS) compared to vemurafenib monotherapy [12.3 vs 7.2 months; hazard ratio (HR) 0.58 (0.46–0.72)]. As part of this study, an interesting biomarker analysis that attempted to link clinical response with baseline oncogenic mutations found no correlation between outcome and either RAS/RAF pathway mutations or tyrosine kinase receptor mutations (RTK) [3]. An update on overall survival (OS) from the Combi-D study of combined dabrafenib plus trametinib in patients with BRAF V600E/K metastatic melanoma was also reported [4]. Patients treated with the combination of dabrafenib and trametinib achieved a median OS of 25.1 months with 51% of patients still alive at 2 years, These findings confirmed results reported from the phase I–II study in 2014 [5]. Finally, data from a phase Ib/II open-label study of patients with BRAFV600-mutant cutaneous melanoma treated with the newer combination of encorafenib plus binimetinib showed an overall response rate (ORR) of 74.5% and a disease control rate (DCR) of 96.4%. Of interest, in the cohort receiving a dosage regimen of encorafenib 400/450 mg and binimetinib 45 mg, the ORR was 77.5% and the DCR was 100%. The combination was also well tolerated, with no grade 3–4 pyrexia or skin toxicity events reported [6]. Data from these three studies are summarized in Table 1.

**Table 1:** Comparison of CR, ORR, PFS, DoR, and OS among the different BRAF and MEK inhibitors combination

BRAFi/MEKi combination	Study	CR (%)	ORR (%)	mPFS (HR)	mDoR	mOS (HR)
Dabrafenib + trametinib	Phase III	13	69	11.0 (0.67)	12.9	25.1 (0.71)
Vemurafenib + cobimetinib	Phase III	15.8	69.6	12.2 (0.58)	12.9	–
Encorafenib + binimetinib	Phase I	12.7	74.5	11.3	–	–

*CR* complete responses, *HR* hazard ratio, *mDoR* median duration of response, *mOS* median overall survival, *mPFS* median progression-free survival, *ORR* overall response rate.

Of even more interest were new data on combination immunotherapy, in particular the randomized, double-blind phase III CheckMate 067 study that compared the combination of nivolumab plus ipilimumab with nivolumab and ipilimumab monotherapies [7]. This study enrolled 945 treatment-naïve patients with advanced disease who were stratified according to PD-L1 expression, BRAF mutation and disease stage. The study was powered to examine differences in PFS and OS for nivolumab or nivolumab plus ipilimumab each versus ipilimumab. PFS data were reported with the combination having a median PFS of 11.5 vs 2.9 months with ipilimumab [HR 0.42 (0.31–0.57)] and 6.9 months with nivolumab [HR 0.57 (0.43–0.76)]. An exploratory analysis showed the combination median PFS to be superior to that of nivolumab monotherapy [HR 0.74 (0.60–0.92)]. In addition, ORR was 57.6% for the combination, 43.7% with nivolumab and 19% with ipilimumab. PFS data stratified by PD-L1 status were especially interesting: with a cut-off of ≥5% for positive PD-L1 expression, median PFS was 14 months for patients treated with either the combination or nivolumab alone compared with 3.9 months in the ipilimumab group. In the PD-L1 negative cohort, the combination confirmed its superiority to both monotherapies with a PFS of 11.2 vs 5.3 months in the nivolumab group and 2.8 months in the ipilimumab group. However, we should be cautious in interpreting these data for several reasons. Firstly, OS data are still awaited, with OS being the best endpoint for immunotherapy. Additionally, in the PD-L1 positive group and in contrast to PFS, ORR was superior with the combination compared to nivolumab monotherapy (72.1 vs 57.5%). Finally, the percentage of patients with positive PD-L1 expression was only 21.7%, −25.3% which is the lowest observed across different studies (Table 2). Even with this stringent cut-off which excluded around two-thirds of responding patients and greatly enriched the cohort with those patients most likely to benefit from nivolumab, the equivalence of single agent to the combination remained unconvincing.

**Table 2:** PD-L1 as a potential biomarker: % of PD-L1 positive patients in different clinical trials

Study	PDL1 positive patients (%)
CA209-037 [21]	49
CA209-066 [22]	35
Ca209-067 ipilimumab/nivolumab arm [7]	21.7
Ca209-067 nivolumab monotherapy arm [7]	25.3
Keynote 006 pembro every 2 weeks [23]	80.6
Keynote 006 pembro every 3 weeks [23]	79.8
Keynote 002 [24]	69
Keynote 001 [25]	77

An important consideration is that the improved PFS and ORR achieved with the combination was accompanied by a high occurrence of side-effects: 55% of patients receiving the combination had grade 3–4 events and 36.4% prematurely discontinued treatment because of its toxicity. However, over two-thirds (67.5%) of patients who discontinued treatment due to toxicities continued to respond. These data are consistent with those observed in another study of combined nivolumab and ipilimumab therapy (CheckMate 069), in which 54% of patients had grade 3–4 adverse events, leading to treatment discontinuation in 38%; 68% of these continuing to respond despite the cessation of treatment [8]. One important characteristic of the immuno-related toxicity associated with the combination was the involvement of more than one organ, which is rare with monotherapy. However, new safety signals were not reported for the combination, with adverse events affecting the same organs as typically seen with monotherapy (i.e. the skin, gastrointestinal tract, liver, endocrine system, lungs). Moreover, these toxicities were manageable using the established algorithm for the treatment of the immuno-related adverse events. Importantly, even in this large multi-national study in which many investigators had not previously used the combination regimen, there were no treatment-related deaths. It should also be noted that these side effects are primarily related to ipilimumab and similar levels of side effects were seen in studies using a high dose of ipilimumab, e.g. the phase III study of first-line combined dacarbazine plus ipilimumab 10 mg/kg (50% grade 3–4 AEs) [9] and in the EORTC adjuvant trial with high-dosage ipilimumab (40.5%) [10].

This high-grade toxicity seen with combined nivolumab and ipilimumab together with the results based on PD-L1 expression has generated the possibility of using the combination in PD-L1 negative patients only, while PD-L1 positive patients might receive nivolumab monotherapy, since this may have a similar impact on PFS with less toxicity. However, as well as taking into account earlier comments on the need to interpret these data with caution, the kinetics of action of the combination and nivolumab alone should also be considered (Figure 1). In comparing data from the phase I trials of nivolumab monotherapy with combined therapy, it is clear that the combination results in an earlier, deeper and more durable response [11, 12]. Moreover, some evidence has even shown a rapid effect of the combination (similar to targeted agents) in patients with bulky disease [13]. As such, assuming the OS data correlates with the ORR data, the combination of nivolumab plus ipilimumab should be considered as the new standard, with the caveat that anti-PD-1 therapy alone may be a valid option in patients where toxicity could be a concern, irrespective of PDL1 status.

**Figure 1:**
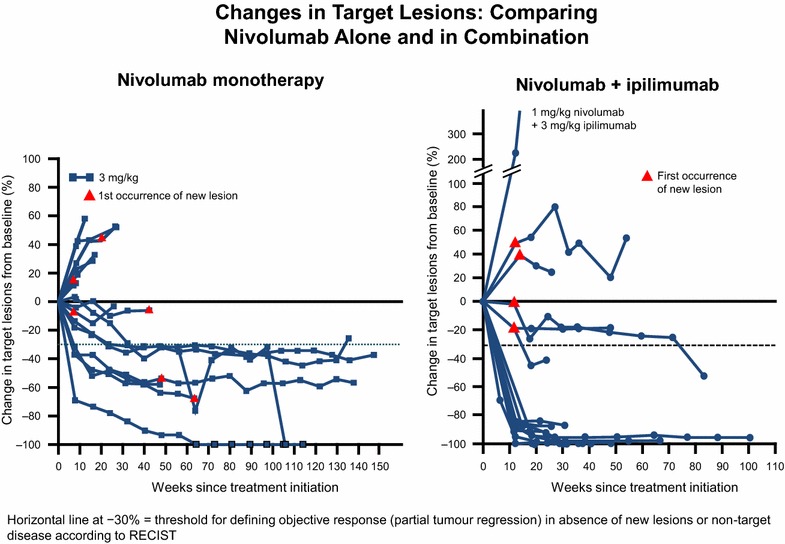
Changes in target lesions: comparison between nivolumab alone (**a**) [11] and in combination with ipilimumab (**b**) [12]. In the phase I studies, the combo ipilimumab/nivolumab showed more rapid and durable changes in target lesions.

Another combined immunotherapeutic approach was the combination of T-VEC (talimogene laherparepvec), an oncolytic virus which includes a gene that encodes for GM-CSF, with ipilimumab [14]. These were an update of data presented at ASCO in 2014 and, in the 18 patients enrolled to date, ORR was 56% and median (PFS) was 10.6 months. Median OS was not reached; 12- and 18-month survival were 72.2 and 67%.

That targeted therapy has an important effect on the immune system is well known and the possibility of combining a BRAF or MEK inhibitor with immunotherapy is an interesting approach. However, phase I data showed that combined vemurafenib and ipilimumab increases liver toxicity (although this was not reported with dabrafenib plus ipilimumab) [15], while the triple combination of ipilimumab plus dabrafenib and trametinib has reported to increase the risk of bowel perforation. The development of anti-PD-1/PD-L1 agents which are more potent and less toxic than ipilimumab means the possibility of a combined approach with a BRAF or MEK inhibitor is more realistic. An interesting phase I study reported data on the combination of the anti-PD-L1 antibody, MEDI4736 (durvolumab) with dabrafenib plus trametinib in patients with stage IIIc/IV melanoma [16]. Patients were enrolled by BRAF status into three different cohorts; BRAF-mutant patients received the triple combination and BRAF wild-type (WT) patients received durvolumab plus trametinib or sequential trametinib then durvolumab. Treatment with the triple combination resulted in an ORR of 69%, and DCR of 100%. In the BRAF WT cohorts, ORR was 21% and DCR was 79% in the combination group, while in the sequential group ORR was 13%, and DCR was 80%; however, data for the sequential group data could be affected by the short-term follow up. Most importantly, these combinations had a manageable safety profile. Despite these promising results, longer follow-up will be necessary to determine the contribution of durvolumab to the impressive activity seen with the triple drug combination.

Finally, emerging data have suggested that predictive markers based on immunoprofiling and mismatch repair deficiency may be more meaningful than PD-L1. The interferon-γ signature 10 gene (related to inflammation) seemed to correlate with a better outcome in patients receiving the anti-PD-1 agent, pembrolizumab, both in terms of PFS and OS [17]. Similarly, although not in melanoma patients, data from a phase I study of patients with renal cell cancer reported that baseline upregulation of genes known to be upregulated by ipilimumab in melanoma, together with other immunorelated genes, was strongly correlated with the outcome [18]. Another important finding was the strong correlation between deficiency in the mismatch repair and the response to immunotherapy that was evidenced in colorectal and other solid cancers and is likely to be a major focus of interest in the future [19].

In conclusion, the use of combination approaches to treat patients with melanoma, as well as other cancers, are no longer a just a wish for the future [20] but are today a clinical reality with a rapidly growing evidence-base. Moreover, the most exciting consideration is that this is far from the end of the story, but rather a fantastic introduction.
